# Accurate and Efficient Detection of Nasopharyngeal Carcinoma Using Multi‐Dimensional Features of Plasma Cell‐Free DNA

**DOI:** 10.1002/hed.28154

**Published:** 2025-04-21

**Authors:** Song Zhang, Jiahui Tang, Pin Cui, Weihuang He, Xiaohui Lin, Shubing Wang, Yuanxian Liu, Xiaohua Tan, Shu Xu, Mingji Feng, Hanming Lai

**Affiliations:** ^1^ Department of Otolaryngology Shenzhen Guangming District People's Hospital Shenzhen China; ^2^ Shenzhen Rapha Biotechnology Incorporate Shenzhen China; ^3^ Department of Oncology People's Hospital of Shenzhen Baoan District, The Second Affiliated Hospital of Shenzhen University Shenzhen China; ^4^ Department of Oncology, Shenzhen Key Laboratory of Gastrointestinal Cancer Translational Research Cancer Institute, Peking University Shenzhen Hospital, Shenzhen‐Peking University‐Hong Kong University of Science and Technology Medical Center Shenzhen China; ^5^ Department of Otolaryngology Shenzhen Traditional Chinese Medicine Hospital Shenzhen China; ^6^ Department of Oncology Shenzhen Third People's Hospital Shenzhen China; ^7^ Department of Oncology Shenzhen Guangming District People's Hospital Shenzhen China

**Keywords:** cell‐free DNA, copy number variations (CNV), machine learning, nasopharyngeal carcinoma, whole genome sequencing (WGS)

## Abstract

**Background:**

The incidence of Nasopharyngeal carcinoma (NPC) is rising in recent years, especially in some non‐developed parts of the world. Hence, cost‐efficient means for sensitive detection of NPC are vital.

**Methods:**

We recruited 646 participants, including healthy individuals, patients with benign nasopharyngeal diseases, and NPC patients for plasma cell‐free DNA(cfDNA), which underwent low‐depth whole‐genome sequencing (WGS) to extract multi‐dimensional molecular features, including fragmentation pattern, end motif, copy number variation(CNV), and transcription factors(TF). Based on these features, we employed a machine learning algorithm to build prediction models for NPC detection.

**Results:**

We achieved a sensitivity of 95.8% and a specificity of 99.4% to discriminate NPC patients from healthy individuals.

**Conclusions:**

This study can be a proof‐of‐concept for these multi‐dimensional molecular features to be implemented as a noninvasive approach for the detection and even early detection of NPC.

## 1 Introduction

Nasopharyngeal cancer (NPC) is a rare type of head and neck cancer, but its incidence is rising in recent years, commonly occurring in southern China, Southeast Asia, and North Africa [1]. China alone witnesses over 60 000 new cases of diagnosed NPC each year, accounting for 1.3% of solid tumors [2]. It is ubiquitously associated with EBV (EBV) infection [3, 4]. Currently, the clinical detection methods for NPC often involve serological testing for EBV. However, the clinical application of NPC screening was hindered by the low positive predictive value, which was only 4.8%, even in the regions where NPC is endemic [5]. Therefore, the development of accurate means of NPC detection, especially early detection, is necessary and urgent.

Plasma cfDNAs are DNA molecules released into the blood circulation from cell deaths originating from surrounding tissues, either healthy or diseased. The cfDNA released by tumor cells is also called circulating tumor DNAs (ctDNAs), which make up only a portion of the mixture of cfDNA from all kinds of cells. The earlier the stage of the tumor is, the lower the abundance of ctDNA in total cfDNA is, which makes the ctDNA abundance for early stage cancer patients typically 0.01% ~ 1%. The most commonly used biomarkers for cancer diagnosis and prognosis are alterations of DNA sequences, including point mutations (e.g., SNP and Indels) and structure variations (e.g., gene fusions). However, in such low ctDNA abundance as mentioned above, sequence alterations in ctDNA are in extremely low ratios and are technically challenging to detect. Besides mutation, epigenetic alterations are changes to epigenetic modifications of genomes to alter regulations of gene expression and have been proven to be effective in the diagnosis and monitoring of various types of solid tumors in recent decades. Epigenetic biomarkers arise in the early stage of carcinogenesis and exist widespread in the whole genome [6]; thus, such biomarkers have been widely used in the early detection of cancers [7, 8]. Among the multiple epigenetic biomarkers, the cfDNA fragmentomic profile has been reported by numerous studies to be used for early detection of several types of common cancers in recent years, for example, lung cancer [9] and colorectal cancer [10], based on the discrepant fragmentomic profiles between plasma cfDNA samples from cancer patients and non‐cancer individuals. The diversity of the composition of different end motifs, stretches of sequences at the ends of cfDNA molecules typically four nucleotides long, was found to be associated with carcinogenesis; for example, the abundance of motif CCCA in HCC patients is significantly lower than that in healthy people [11].

Copy number variations (CNV) have been reported to be a major cause of structural variation in cancer genomes [12]. Compared to point mutations, CNV causes the gain and loss of genomic segments usually at large scale, leading to the change of ctDNA abundance in cfDNA [13], which can be easier to detect; compared to methylation markers, CNV is less prone to be affected by interfering factors like age or lifestyle [14]. Furthermore, numerous studies have reported the association between the expression profile of transcription factor (TF) and complex diseases [15, 16]. TF expression can be an indicator for cancer risk prediction [17] and cancer subtyping [18]. In general, a multi‐dimensional biomarker‐based cfDNA test is becoming a promising means for cancer detection. However, such means have not yet been implemented for NPC detection; thus, we performed this proof‐of‐concept study.

## 2 Materials and Methods

### 2.1 Pipeline and Participants

**FIGURE 1:**
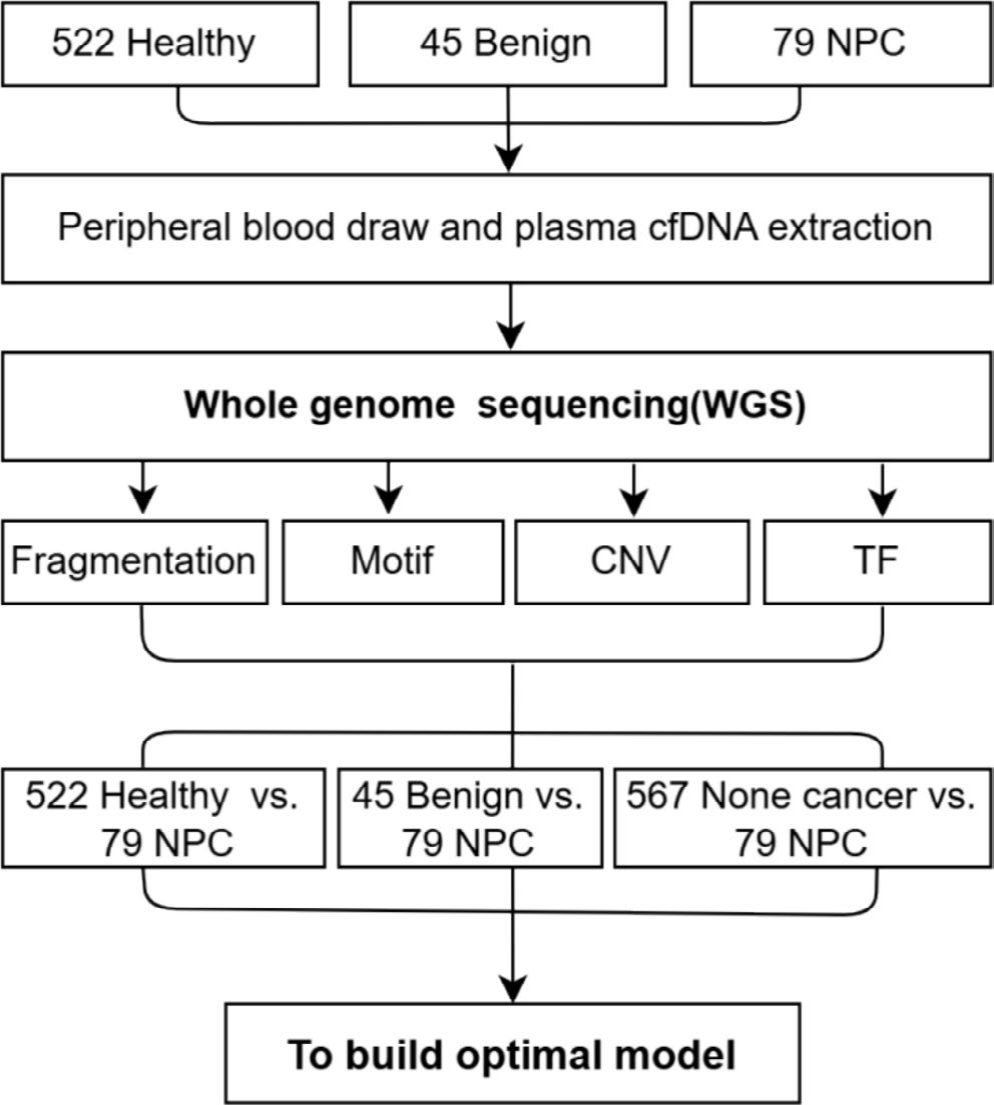
General workflow of this study.

We recruited a total of 646 participants for this study, including 522 individuals for the healthy cohort, 45 patients with benign nasopharyngeal disease for the benign patients' cohort and 79 patients with NPC for cancer cohort. All benign nasopharyngeal and NPC patients were enrolled at the time of diagnosis in the Shenzhen GuangMing District People's Hospital and Shenzhen Traditional Chinese Medicine Hospital. In addition, the healthy individuals were enrolled in the Peking University Shenzhen Hospital. All procedures involving human participants were performed following the Declaration of Helsinki. The protocol obtained review and approval from the Ethics and Scientific Committee of the Shenzhen Traditional Chinese Medicine Hospital (No. (2018)58), Peking University Shenzhen Hospital (NO. 2021‐075), and Shenzhen GuangMing District People's Hospital (NO. LL‐KT‐2022053). We performed whole genome sequencing (WGS) to plasma cfDNA of all these participants. Then we extracted cfDNA fragment size, motif features, CNV, and TF from the WGS data, and we used these features to build prediction models. The models implement a back‐propagation algorithm based on the Adam (Adaptive Moment Estimation) optimizer following training and validation on the basis of previous data features (Figure 1).

### 2.2 Cell‐Free DNA Extraction From Plasma Samples

For each of the 646 participants, we collected 6–10 mL peripheral blood into a cfDNA preservation tube (cat. 20 092 421, Hebei Xinle Medical Instrument Technology Inc., Xinle, China) and shipped the tubes at room temperature to the Molecular Genetics Laboratory (Shenzhen Rapha Biotechnology Inc., China) for cfDNA extraction within 72 h. To obtain plasma, whole blood was centrifuged at 1600 × g for 10 min, from which the supernatant was transferred to a new tube and further centrifuged at 10000 × g for 15 min to remove cell debris from the plasma. For each participant, cfDNA was isolated and purified from 3 mL plasma using the HiPure Circulating DNA Midi Spin Kit S (Magen Biotech Inc., Guangzhou, China) into a final elution volume of 50 μL. Quality control was performed on these libraries using Qsep100 (Bio‐optic. Inc., Taiwan, China) for fragment size distribution and Qubit 4.0 (Thermo Fisher Inc., MA, USA) for concentration, and cfDNA samples with abnormal fragment size distribution (showing distribution outside the normal cfDNA peak) and ultrahigh concentration were identified as contaminated with genomic DNA (mainly from dead white blood cells during logistics). No genomic DNA contamination was observed in each sample of the 646 participants; therefore, the data was used for subsequent analysis.

### 2.3 Whole‐Genome Sequencing Library Construction and Sequencing

We performed whole genome sequencing (WGS) to 10 ng cfDNA of each of the 646 participants. WGS libraries were constructed using the RainbowOne Universal DNA Library Prep Kit for MGI (Shenzhen Rapha Biotechnology Inc., China) following basic principles for NGS library preparation, including molecular end repair, sequencing adaptor ligation, and library clean‐up. The libraries were then amplified using VAHTS HiFi Amplification Mix (cat. N616‐01) and purified using VAHTS DNA Clean Beads (cat. N411‐02), both provided by Vazyme Biotech Co. Ltd., Nanjing, China. Quality control was performed on these libraries using Qsep100 (Bio‐optic. Inc., Taiwan, China) and Qubit 4.0 (ThermoFisher. Inc., MA, USA), and libraries were sent for sequencing in batches of 16, each batch on a separate lane of an MGI‐2000 sequencer(BGI Genomics Inc., Wuhan, China) using DNBSEQ technology and PE100 sequencing mode.

### 2.4 Fragmentation Profile and End Motif Feature

For analysis of 646 samples of WGS data, raw sequencing data were filtered by fastp [19] as part of the quality control protocol. The filtered reads were then mapped onto the human reference genome (GRCh38/UCSC hg38) using the sequence aligner BWA [20], and PCR duplicates were marked by SAMtools [21]. The fragment size for every read pair with a mapping quality score above 30 for either read was extracted from every sample. As suggested by previous studies, short fragments were defined as having lengths between 100 and 150 bp, while the long fragments were defined as having lengths between 151 and 220 bp [22, 23]. The fragmentation pattern was generated using the short/long fragment ratio, as previously described in the DELFI approach [24]. The ratios of the short/long fragments for each sample were examined in 5 Mb bins, resulting in a total of 472 features from the 472 bins genome‐wide after excluding the regions in Duke blacklisted or with low mapping ratios. All 472 fragment features were then used as the input to build the prediction model for Nasopharyngeal carcinoma detection.

To investigate the end motif feature of cfDNA molecules, the first four bases of the R1 end of each cfDNA fragment were used as motif codes, and the proportion of 4^4^ motif codes in each sample was counted as motif features to generate a total of 256 dimensions of features.

### 2.5 Analysis of CNV

To investigate the genome‐wide copy number variation profile, the human reference genome (GRCh38) was tiled into non‐overlapping 0.5‐Mb bins, totaling 4,915 bins.

We used the readCounter module of HMMcopy to count the coverage reads value of aligned bam file against each bin, which were then used for CNV analysis using the R package ichorCNA [25]. NPC content of each bin was corrected by LOESS smoothing over the NPC spectrum, and coverage of each bin was normalized. The depth of each bin was used to compare against the software baseline and compute the log2 ratio.

### 2.6 Coverage Profiling of Transcription Factor Binding Sites

We investigated the transcription factor binding sites (TFBS) by obtaining the data from Gene Transcription Regulation Database (GTRD) in individual BED files per TF. Considering the generally huge numbers of TFBSs for each TF, only the TFs with more than 1000 TFBSs were chosen for subsequent analysis. Then we recalculated the positions by focusing on the reported point with the highest ChIP‐seq signal in the meta‐cluster, and we extracted the top 1000 sites that were supported by most of the analyzed samples, namely 1000‐msTFBSs. Finally, we calculated the coverage profile within the TFBS ±1000 bp region and normalized it using CNV and average coverage, yielding the average coverage depth for every position surrounding the TFBS.

### 2.7 Prediction Models for NPC Detection

Machine learning algorithm XGBoost was employed to build prediction models, with 150 estimators, max depth 2, and learning rate 0.1 as parameters for model training. Meanwhile, to minimize the effect of the difference in the number of participants in the three cohorts on the accuracy of the prediction models, we set the parameter scale_pos_weight as the number of participants in cohort 1 versus the number of participants in cohort 2 while building the models using xgb.XGBClassifier. The participants from each cohort were randomly assigned into a training set and validation set at a 7:3 ratio following the workflow as described in Figure 1.

And the performance of prediction models is evaluated using the fundamental statistical parameters, which are sensitivity, specificity, accuracy, true positive rate (TPR) and false positive rate (FPR). Then TPR and FPR were used to draw the receiver operating characteristic (ROC) curve, yielding the value of the area under the ROC curve(AUC), as described in previous studies [10, 26].

## 3 Result

### 3.1 cfDNA Fragmentation Profiles of Three Cohorts

**FIGURE 2:**
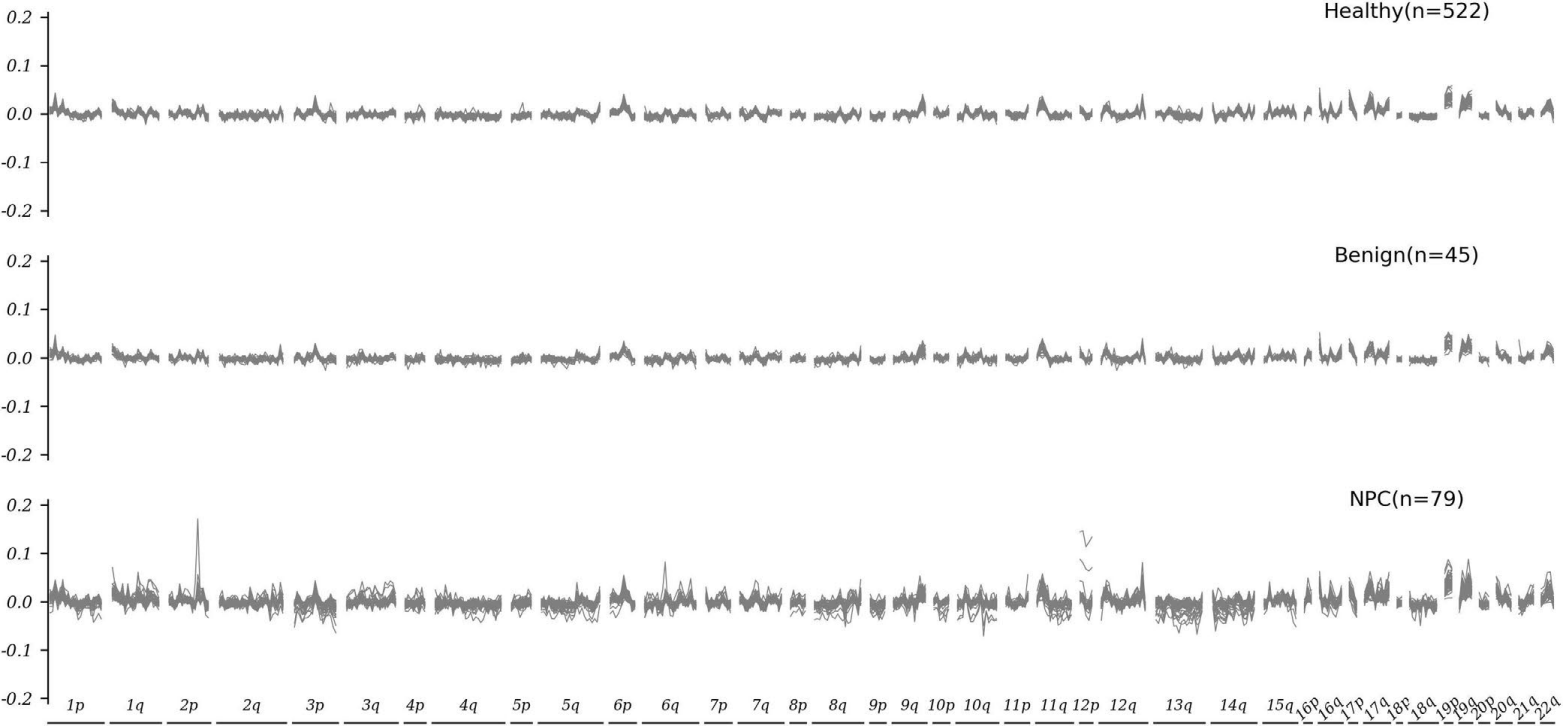
Fragmentation profiles of cfDNA samples from different cohorts. Fragmentation profiles of cfDNA samples from healthy cohort, nasopharyngeal benign disease cohort, and NPC cohort. The X‐axis represents 5 Mb bins across the human genome, while the Y‐axis represents the difference to the average ratio of short to long cfDNA fragments for each bin.

All 646 participants recruited in this study underwent WGS and data analysis, including QC and mapping, yielding an average sequencing depth of 1.13x and an average map‐rate of 91.37% (Table S1), which is within the acceptable range (0.5x–5x) of cancer detection using low depth WGS as suggested by a previous study [27]. For visualization of the cfDNA fragmentation profiles, we plotted the fragment feature of the bin minus the average fragment feature of all 472 bins on the Y‐axis, while the X‐axis marks the order and location of all 472 bins by chromosome (Figure 2). Generally, the profiles of the NPC cohort showed much stronger fluctuations than those of the healthy cohort, while the fluctuation of the curves of both the healthy cohort and the nasopharyngeal benign disease cohort was limited to a narrow range.

### 3.2 End Motifs of cfDNA

**FIGURE 3:**
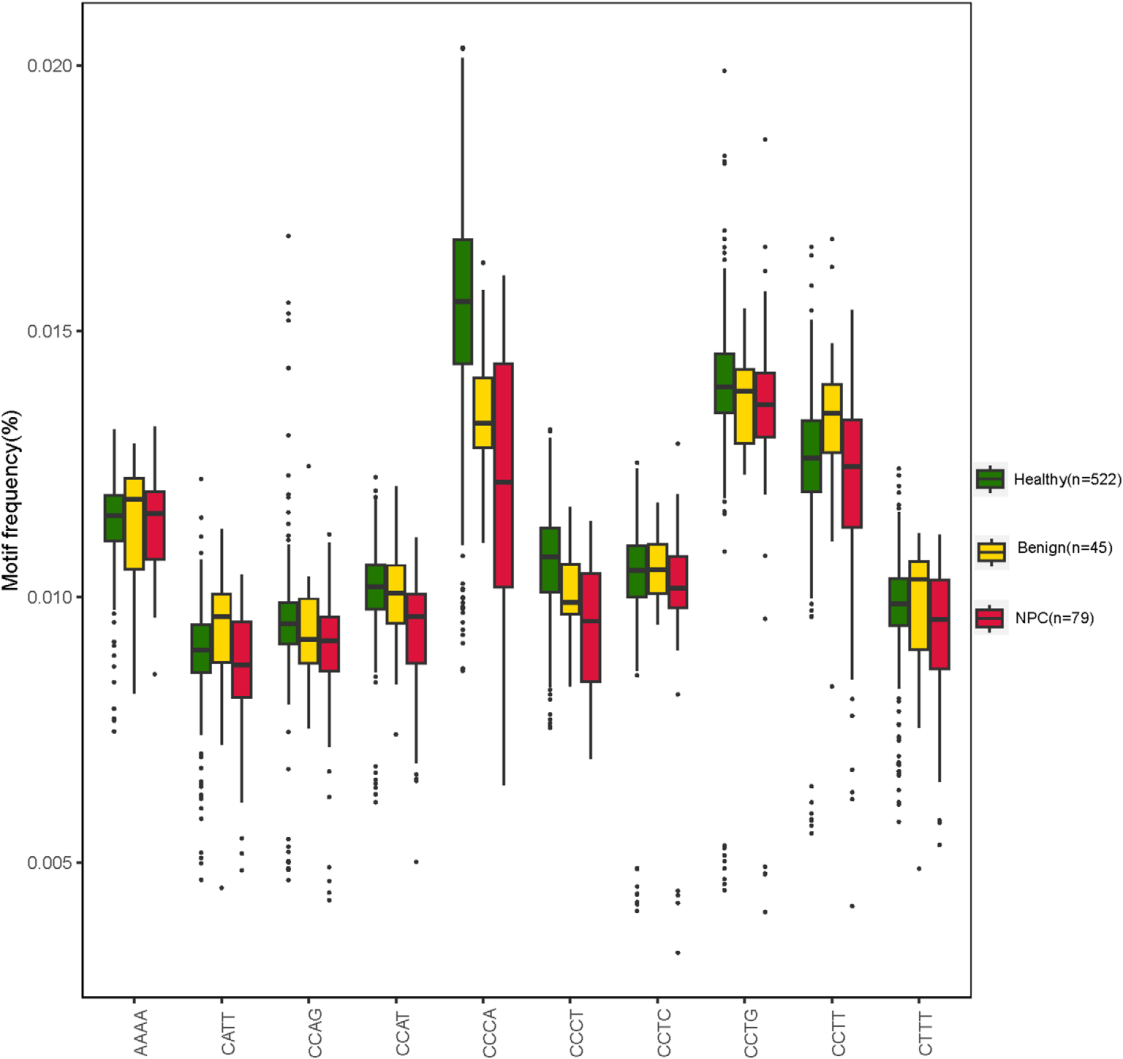
Motif feature of three cohorts on the top 10 selected motifs by proportion. The abscissa represents the motif sequence, and the ordinate represents the proportion of each of the 10 motifs. [Color figure can be viewed at wileyonlinelibrary.com]

From the same WGS data, molecular end motif features were extracted for all 646 participants and sorted into three cohorts (Table S1). The overall end motif features of the three cohorts were compared, showing discrepancies between the values of the three groups, especially among the top 10 selected motifs by proportion (Figure 3). Generally, the motif feature values of healthy individuals were the highest, while the NPC patients were the lowest. Therefore, 256 motif eigenvalues can be identified (Figure S1), which can be used as markers to build prediction models.

### 3.3 Plasma cfDNA CNV Profiling

**FIGURE 4:**
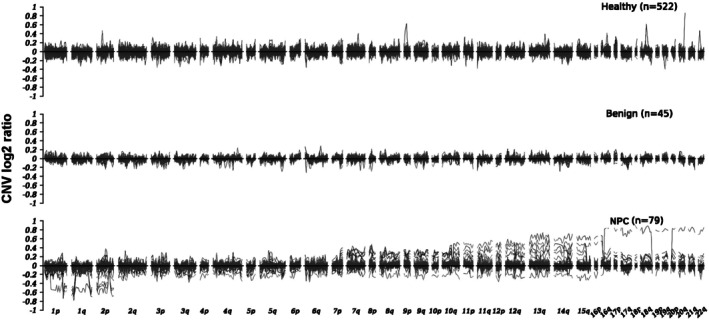
CNV profiles of cfDNA samples from different groups. The X‐axis represents 0.5 Mb bins across the human genome, while the Y‐axis represents the CNV log2 ratio values in each bin.

By comparing CNV features of different groups (Figure 4), we found that CNV values in the NPC group exert substantial fluctuations across most chromosomes, while the fluctuations in the CNV profiles of healthy and benign groups are quite limited. In the chromosomal regions 1p–2p, the CNV profile showed a trend of loss. The CNV profile in chromosomal regions 2q–7p exhibited both gain and loss, while the profile of chromosomal regions 7q–16p showed gain and loss with a higher proportion for CNV gain. Furthermore, in chromosomal regions 16q–22q, CNV variations showed a trend of gain. Generally, CNV can potentially be a biomarker for NPC detection.

### 3.4 TFBS Coverage Profiling

The WGS coverage depth TFBS is directly associated with the level of the binding of transcription factors(TFs) to the TFBS in chromatin, as only the TF‐bound DNA can avoid being degraded by the exonuclease in the blood and ultimately retain as cfDNA for our WGS assay. As a fundamental principle in biology, the expression of a gene is normally initiated by the binding of TF to TFBS; thus, the altered binding profile of TF can cause abnormal gene expression, for example, the up‐regulated expression of oncogenes and down‐regulated expression of tumor‐suppressor genes, hence the initiation of tumors. Therefore, the TFBS coverage profile in this WGS‐based assay is associated with the carcinogenesis of NPC.

**FIGURE 5:**
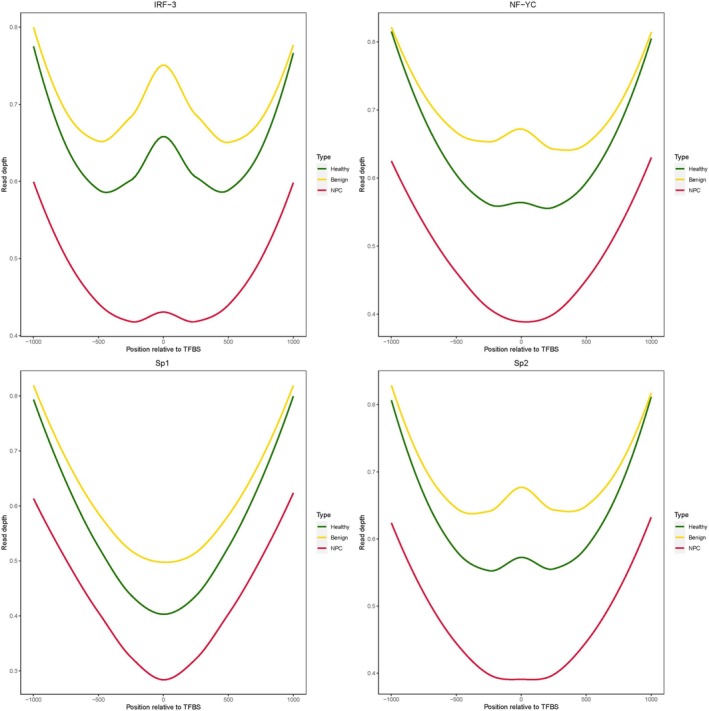
TFs analysis. The read depth of TFBSs in the healthy cohort (curves in green), nasopharyngeal benign disease cohort (curves in yellow), and NPC cohort (curves in red). [Color figure can be viewed at wileyonlinelibrary.com]

We compared the coverage profiles of TFBS of cfDNA samples of the three cohorts (Figure 5) and found that the coverage depth of the NPC cohort is significantly lower than that of the healthy cohort in the whole region of downstream 1000 bp and upstream 1000 bp of TFBSs. In contrast, the coverage depth of the benign diseases cohort is much higher than that of the healthy cohort in a narrower region of downstream 500 bp and upstream 500 bp of TFBSs. Meanwhile, certain TFs, including IRF3 [28], NFYC [29], SP1 [30], and SP2 [31], have been reported to be strongly associated with the genesis and progress of NPC. Some TFs exert different functions in different conditions, either stimulating the expression of oncogenes or down‐regulating the expression of tumor‐suppressor genes. For instance, in the Toll‐like receptor 4 (TLR4) activated micro‐environment of NPC, IRF3 and NF‐kB both participate in the nucleosome remodeling, hence the up‐regulation of oncogene transcription. Through binding to the specific enhancer regions, IRF3 elevates the chromatin accessibility, thus promoting the expression of downstream oncogenes like PD‐L1 [32], which has been found in the early stage of tumoriogenesis in the nasopharynx before going malignant. Additionally, as a key downstream factor for the cGAS‐STING pathway [33], IRF3 can inhibit the progress of EBV‐related NPC by stimulating the anti‐viral immune response. Therefore, we propose that coverage profiles of TFBS of cfDNA could be a biomarker for NPC detection.

### 3.5 Prediction Models to Detect NPC Using cfDNA Multi‐Dimensional Biomarkers

**FIGURE 6:**
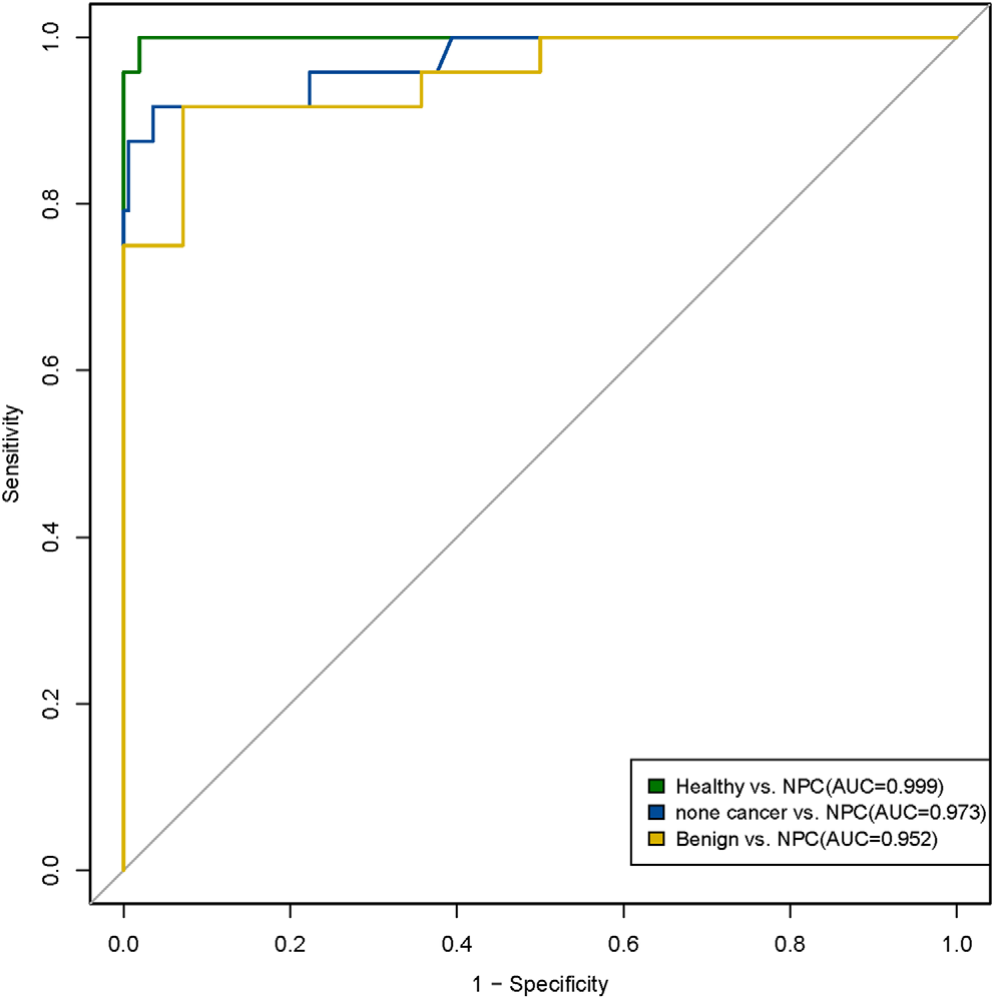
Machine learning models detect NPC patients with high sensitivity and specificity based on cfDNA fragmentation profiles. Green ROC curve represents the prediction model to discriminate NPC patients from healthy individuals. Blue ROC curve represents the prediction model to discriminate NPC patients from non‐cancer individuals. Yellow ROC curve represents the prediction model to discriminate NPC patients from benign nasopharyngeal diseases. [Color figure can be viewed at wileyonlinelibrary.com]

To discriminate between healthy individuals, benign patients, and NPC patients, 646 samples were used for modeling; healthy individuals and patients with benign NPC are defined as the non‐cancer group. The AUC of the prediction model for healthy versus NPC was 0.999, with a sensitivity of 95.8% and a specificity of 99.4%. The AUC of the prediction model for non‐cancer versus NPC was 0.973, with a sensitivity of 87.5% and a specificity of 99.4%. The AUC of the prediction model for Benign versus NPC was 0.952, with a sensitivity of 91.7% and a specificity of 92.9%. The AUC of each group of models is shown in Figure 6. Furthermore, the positive detection rate of our prediction models is higher than the performance of the EBV serological test [5], the positive predictive value of which was only 4.8%. Overall, these results suggest that multi‐dimensional biomarkers may potentially become a noninvasive assay for the detection and stratification of NPC.

## 4 Discussion

Various means for detection of NPC have been explored worldwide, including nasopharyngoscopy, biopsy, EBV virus serological testing, and magnetic resonance imaging (MRI) of the nasopharynx and neck [34]. Regrettably, these methods were not able to achieve the accuracy and cost‐efficiency sufficient for early detection of NPC in a widespread fashion.

During the past decade, plasma cfDNA has been utilized as an effective approach for cancer diagnosis and monitoring [35]. In this study, we collected plasma cfDNA multi‐dimensional biomarkers from 646 participants, including healthy individuals, benign nasopharyngeal patients, and NPC patients. Then, based on these multi‐dimensional biomarkers, we built automatic prediction models for NPC detection using a machine learning algorithm. The AUC of the Healthy versus NPC model is 0.999, which obviously outperforms EBV serological testing [36, 37]. In technical aspects, all data were obtained only through low‐pass WGS (approximately 1.13×) in our study, which maintains low cost and labor consumption compared to MRI. Additionally, the sampling approach of this study was blood draw rather than Nasopharyngoscopy and MRI, which greatly increases compliance in real‐world practice, especially for screening NPC in a large population.

Furthermore, to test if our method could discriminate all non‐cancer participants from NPC patients, we combined the healthy cohort and the patient cohort with nasopharyngeal benign diseases together to form a non‐cancer group to be marked as negative against the NPC patient cohort marked as positive. This prediction model also achieved remarkable performance with a sensitivity of 87.5% and specificity of 99.4%, providing enough precision for clinical application. However, the numbers of samples in the benign diseases cohort and NPC cohort are relatively small, so there is still much room for improvement in the accuracy of our assay if optimized in a larger sampling size of retrospective study and better in a prospective study in the future, as the performance of machine learning‐based classifiers relies on sampling size for learning.

Remarkably, our method is based on merely low‐depth WGS, which provides a cost‐efficient means for NPC screening in large‐size population. Overall, this study has revealed clearly different profiles of cfDNA multi‐dimensional biomarkers of the NPC cohort compared to those of the healthy cohort or nasopharyngeal benign cohort, including the cfDNA fragmentomic, motif, CNV, and TF. Using machine learning‐based in silico classifiers, NPC detection achieved considerable accuracy at the cost of only low‐depth WGS. Hence, this study provides preliminary proof for a non‐invasive and cost‐efficient approach for the accurate detection of NPC.

## Author Contributions

**Song Zhang:** conceptualization, supervision, project management, manuscript revision. **Jiahui Tang:** investigation, resources, manuscript revision. **Pin Cui:** conceptualization, methodology, supervision, funding acquisition, writing original draft. **Weihuang He:** methodology, validation, writing original draft. **Xiaohui Lin:** data curation, formal analysis, resources, manuscript revision. **Shubing Wang:** data curation, formal analysis, resources, funding acquisition. **Yuanxian Liu:** data curation, formal analysis, resources. **Xiaohua Tan:** data curation, formal analysis, resources. **Shu Xu:** data curation, formal analysis, resources. **Mingji Feng:** methodology, validation, formal analysis. **Hanming Lai:** validation, formal analysis.

## Ethics Statement

The protocol obtained review and approval from the Ethics and Scientific Committee of the Shenzhen Traditional Chinese Medicine Hospital (No. (2018)58), Peking University Shenzhen Hospital (No. 2021‐075), and Shenzhen GuangMing District People's Hospital (No. LL‐KT‐2022053). All participants provided written informed consent for the scientific use of their clinical data and samples.

## Consent

The authors have nothing to report.

## Conflicts of Interest

The authors declare no conflicts of interest.

## Supporting information


**Figure S1.** Motifs.

**Table S1.** Mapping QC of all WGS data in this study.

## Data Availability

Data sharing not applicable to this article as no datasets were generated or analysed during the current study.
