# Supplementary figures and images for: Accurate and Efficient Detection of Nasopharyngeal Carcinoma Using Multi‐Dimensional Features of Plasma Cell‐Free DNA
Source: Head Neck. 2025 Apr 21;47(9):2499–506. doi: 10.1002/hed.28154 (PMC12338027; doi:10.1002/hed.28154)

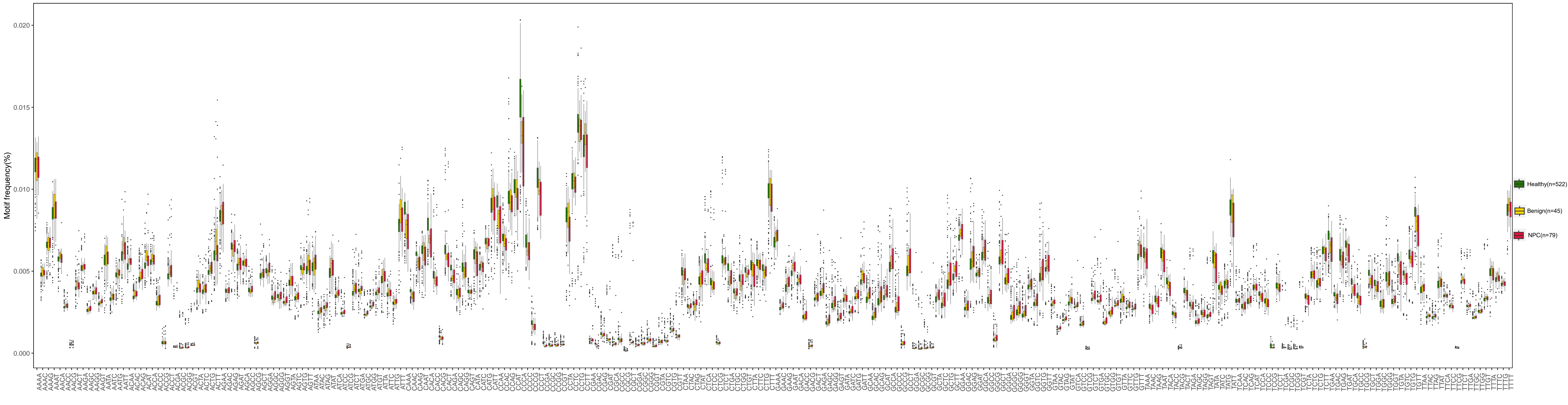

Supplement: Supplementary file 1 — Figure S1. Motifs. [file HED-47-2499-s002.pdf]
